# A multicenter randomized controlled trial of a plant-based nutrition program to reduce body weight and cardiovascular risk in the corporate setting: the GEICO study

**DOI:** 10.1038/ejcn.2013.92

**Published:** 2013-05-22

**Authors:** S Mishra, J Xu, U Agarwal, J Gonzales, S Levin, N D Barnard

**Affiliations:** 1 Clinical Research, Physicians Committee for Responsible Medicine, Washington, DC, USA; 2 Department of Medicine, George Washington University School of Medicine and Health Sciences, Washington, DC, USA

**Keywords:** plant-based, vegan, vegetarian, weight loss, clinical trial

## Abstract

**Background/objectives:**

To determine the effects of a low-fat plant-based diet program on anthropometric and biochemical measures in a multicenter corporate setting.

**Subjects/methods:**

Employees from 10 sites of a major US company with body mass index ⩾25 kg/m^2^ and/or previous diagnosis of type 2 diabetes were randomized to either follow a low-fat vegan diet, with weekly group support and work cafeteria options available, or make no diet changes for 18 weeks. Dietary intake, body weight, plasma lipid concentrations, blood pressure and glycated hemoglobin (HbA_1C_) were determined at baseline and 18 weeks.

**Results:**

Mean body weight fell 2.9 kg and 0.06 kg in the intervention and control groups, respectively (*P*<0.001). Total and low-density lipoprotein (LDL) cholesterol fell 8.0 and 8.1 mg/dl in the intervention group and 0.01 and 0.9 mg/dl in the control group (*P*<0.01). HbA_1C_ fell 0.6 percentage point and 0.08 percentage point in the intervention and control group, respectively (*P*<0.01).

Among study completers, mean changes in body weight were −4.3 kg and −0.08 kg in the intervention and control groups, respectively (*P*<0.001). Total and LDL cholesterol fell 13.7 and 13.0 mg/dl in the intervention group and 1.3 and 1.7 mg/dl in the control group (*P*<0.001). HbA_1C_ levels decreased 0.7 percentage point and 0.1 percentage point in the intervention and control group, respectively (*P*<0.01).

**Conclusions:**

An 18-week dietary intervention using a low-fat plant-based diet in a corporate setting improves body weight, plasma lipids, and, in individuals with diabetes, glycemic control.

## Introduction

Approximately two-thirds of Americans are currently overweight, half of whom are obese.^1^ Obesity is associated with increased risk of serious health conditions, including type 2 diabetes, cardiovascular disease, hypertension and certain cancers that account for about 75% of the $2 trillion spent on medical care each year.^2^

The workplace is an ideal location for nutritional interventions. It is where many individuals make dietary choices, receive health information and spend much of their day. Employers have an economic interest in employee health, particularly given that obesity is associated with increased use of sick leave and disability expenditures,^3^ reduced job productivity and increased absenteeism.^4^ In a 2010 study, the total cost of obesity in the workplace was estimated to be $73.1 billion, 41% of which could be attributed to reduced productivity, 18% to absenteeism and 41% to medical expenditures.^4^

A prior study of a dietary intervention involving two corporate sites of the Government Employees Insurance Company (GEICO), a major US insurance company with about 27 000 employees nationally, demonstrated that a low-fat plant-based diet led to favorable changes in body weight, plasma lipid concentrations and glycemic control.^5^ A plant-based diet was selected because studies had shown that people following vegetarian and near-vegetarian diets have significantly lower prevalence of obesity,^6, 7^ type 2 diabetes,^8, 9^ heart disease,^10^ hypertension,^11^ cancer^12^ and gallbladder disease,^13^ compared with non-vegetarians. In clinical trials, low-fat plant-based diets reduce body weight and blood pressure, and improve plasma lipid concentrations and glycemic control.^14, 15^

We conducted the present multicenter study to evaluate the effects of a low-fat plant-based diet on health outcomes in a larger and more geographically diverse sample of GEICO employees.

## Materials and methods

### Study population

Men and women >18 years of age with a body mass index (BMI) ⩾25 kg/m^2^ and/or a previous diagnosis of type 2 diabetes were recruited through advertisements and group meetings at 10 GEICO corporate offices encompassing over 20 000 employees, in Tucson, Arizona; San Diego, California; Lakeland, Florida; Macon, Georgia; Chevy Chase, Maryland; Buffalo, New York; Woodbury, New York; Dallas, Texas; Fredericksburg, Virginia and Virginia Beach, Virginia. Exclusion criteria included current alcohol or drug abuse, pregnancy, history of severe mental illness, unstable medical status, current adherence to a low-fat, vegetarian diet, participation in the previous GEICO two-site study and inability to attend weekly meetings. Worksites were then pair-matched by race and each pair of sites represented a cluster. The sites within each pair (cluster) were randomly assigned to the intervention group (five sites) or control group (five sites) using a random-number table. As assignment was done by site rather than by individual, all participants at a given site were in the same assigned group. The study was approved by an external institutional review board, and all participants provided written informed consent.

### Intervention program

**Figure 1:**
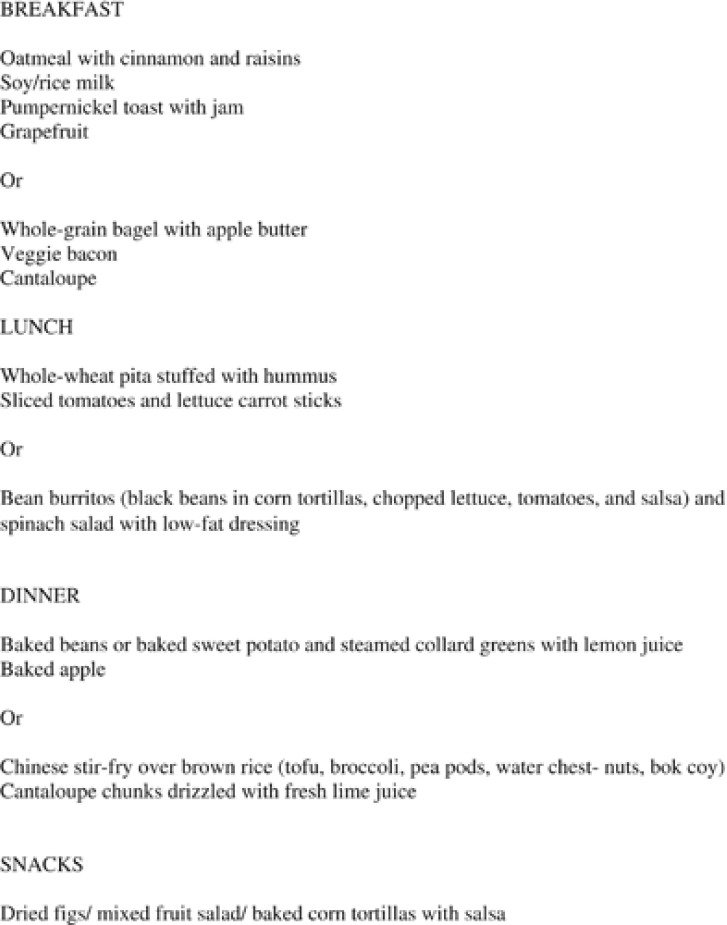
Low fat vegan menu sample.

Participants at intervention sites were asked to follow a low-fat vegan diet consisting of whole grains, vegetables, legumes, and fruits, with no restriction on energy intake for 18 weeks. They were asked to avoid animal products (that is, meat, poultry, fish, dairy products and eggs) and to minimize added oils, with a target of <3 g of fat per serving. They were also encouraged to favor foods with a low glycemic index, which have been shown to decrease triglyceride concentrations^16^ and increase insulin sensitivity independent of effect on body weight.^14, 17^ Intervention group participants were asked to take a daily supplement of vitamin B12. At intervention sites with cafeterias, low-fat vegan menu options were made available, such as oatmeal, minestrone or lentil soup, veggie burgers and portobello mushroom sandwiches among the daily offerings. The low-fat vegan menu options were highlighted in the cafeteria, but the daily vegan options varied depending on the individual cafeterias. A sample of the menu followed by the participants in the intervention group is shown in Figure 1.

The participants at the intervention sites were asked to follow a low-fat vegan diet for 18 weeks. They were provided group support in a total of 18, weekly lunch-hour classes held at the worksite for the duration of the study. The classes were led by a registered dietitian, physician and/or a cooking instructor. All instructors received training in study procedures and followed predetermined identical instruction materials (curriculum, handouts, videos, cooking instructions, and so on). Classes included nutrition education lecture videos on topics such as the effects of diet on weight loss, diabetes, heart disease and cancer, as well as cooking demonstrations and group discussion. Individuals at intervention sites were not compensated.

Individuals at control sites made no dietary changes, were given no dietary guidance and no additional food was made available in those sites. They were given $50 gift certificates for completion of all aspects of the study.

All participants were asked not to alter their exercise patterns during the study period. Participants were asked to continue their pre-existing medication regimens unless modified by their personal physician. No restrictions were placed on use of medications during the study. Information on current medication use was collected at baseline and week 18.

### Measurements

The following measures were assessed at weeks 0 and 18:

A 24-h diet recall was used to assess nutrient intake over two 24-h periods, using an online program (ASA24; Automated Self-Administered 24-hour Recall) developed by the National Cancer Institute, Bethesda, MD, USA (http://riskfactor.cancer.gov/tools/instruments/asa24/).

The format and design of the online program were modeled on the interviewer-administered Automated Multiple Pass Method 24-h recall developed by the US Department of Agriculture. Participants with implausible energy intake (<800 or >4000 kcal per day among men and <500 and >3500 kcal per day among women) were excluded from nutrient data analyses.

Body weight was measured with participants wearing light, indoor clothing without shoes, using a digital scale (Befour Inc., Saukville, WI, USA; model number FS-0900, Health & Fitness Stand-on Scale). Height was measured with participants standing barefoot with their backs to a wall-mounted stadiometer and heels against the wall.

Digital blood pressure monitor (Omron Healthcare, Inc., Kyoto, Japan, model number Omron HEM780) was used to measure blood pressure. Three measurements were taken at 1-min intervals. The mean of the last two measurements was calculated.^18^

Blood samples were collected in the morning after a 12-h fast. Plasma cholesterol and triglyceride concentrations were measured using an Abbott Spectrum analyzer (Abbott Laboratories, Abbott Park, IL, USA) by enzymatic methods.^19^ High-density lipoprotein (HDL) cholesterol concentration was measured after double precipitation with dextran and MgCl_2_.^20^ Low-density lipoprotein (LDL) cholesterol concentration was estimated using the Friedewald equation.^21^ In cases where triglycerides exceeded 400 mg/dl, LDL was measured by affinity chromatography. For participants with diabetes, HbA_1C_ was measured by immunoturbidimetric determination (Gen.2, Tina-Quant, Whole Blood Application on the Roche Integra 800, Roche Diagnostics, Basel, Switzerland). All laboratory tests were conducted by Quest Diagnostics (Quest Diagnostics, Madison, NJ, USA).

At the conclusion of the intervention period, individuals in the control group were offered the same series of 18 total dietary instructional sessions as had been provided to the intervention group. Weight, blood pressure, cholesterol, triglycerides and HbA_1C_ were measured before and after an 18-week period for this group, using the methods described above.

### Statistical analysis

A power analysis, using the previous GEICO study showed that an effect size was >0.30 for the following clinical variables (weight, total cholesterol, HDL cholesterol and blood pressure). With the effect size >0.30, α of 0.05 and power of ⩾80%, a sample of 175 subjects in each group was required to detect a statistical significance between the two groups. Assuming a 20% drop out, 210 participants were required per group. If this sample could not be achieved in the initial study, a replication could be considered.

Distributions of variables were examined for skewness. *T*-tests and *χ*^2^ tests identified measures that were unbalanced between groups at baseline so as to be included as covariates in subsequent analyses. As exclusion of outliers did not alter results, results are presented without such exclusions.

An intention-to-treat analysis was performed on dietary and clinical variables, where participants who did not complete 18-week assessments were deemed to have had no change from baseline. Participants who did not complete clinical baseline assessments were not accepted into the study. Within-group changes were determined by paired *t*-tests. Between-group changes were determined by *t*-tests and general linear model univariate analysis (analysis of covariance), where treatment group, gender, cluster and medication changes were included as fixed factors and baseline variables as covariates. *χ*^2^ tests determined whether there were differences between groups regarding the number of participants losing ⩾5% of body weight.

A separate analysis was performed on those participants who completed 18-week assessments to determine the effect of the intervention on clinical variables among program completers. A supplemental analysis was conducted on those participants who did not change their medications during the course of the study.

All analyses were conducted using SPSS version 18.0. (IBM, Armonk, NY, USA) *P*-values <0.05 were considered significant.

## Results

### Study population

Of the 319 participants screened for eligibility, 291 (142 at intervention sites and 149 at control sites) met the participation criteria and were enrolled in the study. Twenty eight participants did not meet the participation criteria and were excluded owing to various reasons, as described in Figure 1. The intervention sites were in Tucson, Macon, Chevy Chase, Lakeland and Buffalo. The control sites were in San Diego, Woodbury, Fredericksburg, Dallas and Virginia Beach. There was no difference in the baseline characteristics among volunteers excluded before randomization and those who were enrolled (*P*-value ranged from 0.1 to 0.8).

**Figure 2:**
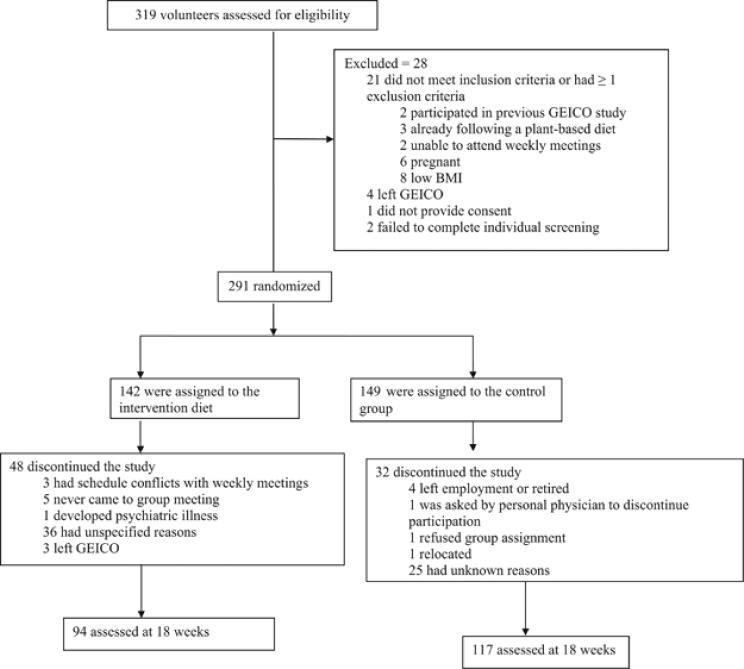
Recruitment and flow of participants through trial.

Ninety-four of 142 (66%) intervention-group participants and 117 of 149 (79%) control-group participants completed anthropometric and laboratory assessments at 18 weeks. Eighty participants dropped out owing to various reasons, as described in Figure 2. At baseline, no significant differences were found between the intervention and control groups for any demographic or clinical measures except gender, with fewer men and more women in the control group (*P*=0.02) (Table 1). There were no intervention-related serious adverse events.

### Dietary intake and adherence

Diet recalls were completed at baseline by 94% of participants in the intervention group and 95% in the control group, and at 18 weeks by 61% of participants in the intervention group and 73% in the control group. Fourteen intervention-group and eight control-group participants had implausible energy intake values at baseline and were excluded from the dietary analysis. At baseline, there were no significant group differences in energy, protein or total fat intake, although there were small differences in carbohydrate and fiber intake (Table 2). Dietary total fat and saturated fat intake were calculated as a gauge of adherence to the diet. Compared with the control group, total fat, saturated fat, protein and cholesterol intakes decreased significantly in the intervention group, while fiber intake increased at 18 weeks. The difference in total and saturated fat intakes was significant between the two groups at 18 weeks (Table 2).

As animal products are the only significant source of dietary cholesterol, cholesterol intake was used as an additional gauge of adherence. Cholesterol intake was ⩽50 mg/day among 47% and 12% of participants in the intervention and the control group, respectively. Total fat intake was ⩽25% of energy among 30% and 6% of participants in the intervention and the control group, respectively. Saturated fat intake was ⩽5% of energy among 29% and 4% of participants in the intervention and the control group, respectively.

### Anthropometric variables

Including all participants in the analysis, mean body weight decreased 2.9 kg in the intervention group and 0.06 kg in the control group (*P*<0.001). BMI fell by 1.04 kg/m^2^ in the intervention group and 0.01 kg/m^2^ in the control group, (*P*<0.001). The between-group differences remained significant after taking into account gender, baseline values, cluster and medication changes (*P*<0.001; Table 3). Weight loss of ⩾5% of body weight was more frequent in the intervention group (37%) compared with the control group (11% *P*<0.001).

Limiting the analysis to the participants who completed the 18-week assessments, mean body weight decreased by 4.3 kg in the intervention group and 0.08 kg in the control group (*P*<0.001). BMI fell 1.5 kg/m^2^ in the intervention group and 0.02 kg/m^2^ in the control group, *P*<0.001. The between-group differences remained significant after adjusting for gender, cluster and baseline values (*P*<0.001; Table 4).

### Plasma lipid concentrations and blood pressure

Including all study participants, changes in total cholesterol were −8.0 mg/dl in the intervention group and −0.01 mg/dl in the control group (*P*<0.01). LDL cholesterol fell 8.1 mg/dl in the intervention group and 0.9 mg/dl in the control group (*P*<0.01). HDL cholesterol decreased 1.8 mg/dl in the intervention group and increased 0.9 mg/dl in the control group (*P*<0.01). Triglycerides increased 9.9 mg/dl in the intervention group and decreased 1.4 mg/dl in the control group (*P*<0.05). These differences remained significant after adjusting for gender, baseline measures, cluster and medication changes (*P*<0.001 for total, LDL and HDL cholesterol; *P*<0.02 for triglycerides). There was no significant difference in the changes in total:HDL cholesterol ratio among intervention- and control-group participants (Table 3).

Analyses limited to those with no medication changes did not substantially differ from those for the full sample (data not shown).

Among participants with baseline triglyceride values <150 mg/dl, triglyceride levels increased 18.1 mg/dl in the intervention group and 6.7 mg/dl in the control group (*P*=0.02). There was no significant difference in the changes in mean triglyceride levels among intervention- and control-group participants with triglycerides ⩾150 mg/dl at baseline.

Limiting the analysis to study completers, total cholesterol fell 13.7 mg/dl in the intervention group and 1.3 mg/dl in the control group, (*P*<0.001). LDL cholesterol fell 13.0 mg/dl in the intervention group and 1.7 mg/dl in the control group (*P*<0.001). HDL cholesterol decreased 3.3 mg/dl in the intervention group and increased 0.7 mg/dl in the control group (*P*<0.01). Triglycerides increased 13.9 mg/dl in the intervention group and decreased 2.9 mg/dl in the control group (*P*<0.05). These differences remained significant after adjusting for gender, baseline measures and cluster. There was no significant difference in the changes in total:HDL cholesterol ratio among intervention- and control-group participants (Table 4).

Systolic and diastolic blood pressures fell slightly in both groups, with no significant between-group differences (Table 3). An analysis limited to medication-stable participants yielded a similar result.

### Hemoglobin A_1c_ in participants with diabetes

Including all participants with type 2 diabetes, mean HbA_1C_ levels decreased 0.6 percentage point and 0.08 percentage point in the intervention and control group, respectively, (*P*<0.01). This difference remained significant after adjusting for gender, baseline measures, cluster and medication changes (*P*=0.004, Table 3). Limiting the analysis to those with no medication changes did not substantially alter the result (data not shown).

Limiting the analysis to participants with type 2 diabetes who completed 18-week assessments, mean HbA_1C_ decreased 0.7 percentage point within the intervention group and 0.1 percentage point in the control group (*P*<0.01). This difference remained significant after adjusting for gender, cluster and baseline measures (*P*=0.003, Table 4).

For those members of the control group who, at the conclusion of the regular study period, began the optional vegan diet program, clinical changes at 18 weeks were as follows. In intention-to-treat analysis (*N*=119), the average reduction in body weight was 3.03 kg (*P*<0.001) and BMI reduced by 1.07 kg/m^2^ (*P*<0.001) at 18 weeks. On average, the total cholesterol was reduced by 3.03 mg/dl (*P*=0.04) and HDL cholesterol by 1.78 mg/dl (*P*=0.003). Limiting the analysis to those who completed both the baseline and 18-week assessments (*N*=94), the average reduction in body weight was 3.84 kg (*P*<0.001) and HDL cholesterol reduced by 2.11 mg/dl (*P*=0.03).

## Discussion

The need for nutrition intervention programs in work settings is driven by both medical and economic concerns. Obesity, diabetes, cardiovascular disease and other diet-related diseases continue to take a major toll worldwide. In the United States, these illnesses exact a financial toll, much of which is borne by employers. An estimated 25–30% of medical costs incurred by employers are attributable to excess risk associated with specific factors, including overweight, heart disease, lipid disorders, diabetes and hypertension, among others.^22^ A previous two-center study suggested that the benefits of plant-based diets seen in clinical settings may also be realized in the workplace. A plant-based dietary intervention was associated with significant weight loss^5^ and was both nutritious^23^ and well accepted.^24^ The present study extends those findings to a larger sample in corporate settings in regions throughout the United States.

In this randomized, controlled trial, a nutrition intervention at the workplace yielded significant improvements in body weight, plasma lipids and glycemic control among diabetics. Changes in these variables in the intervention group were greater than that in the control group, and were statistically and clinically significant. Although many intervention-group participants had less than complete adherence to the prescribed diet, dietary changes were substantial, and significant changes in anthropometric and clinical variables were evident.

The weight changes observed in the present study were similar to those seen with plant-based diets in observational or research settings.^25^ It is noteworthy that a plant-based diet causes weight loss even in the absence of caloric restriction and exercise.^25^ Weight reduction appears to result from early satiety due to higher dietary fiber intake, leading to a drop in energy intake. The difference in weight loss could also be the result of an increase in the thermic effect of food, allowing a small extra edge for weight loss in the vegan group. A low-fat vegan diet increases insulin sensitivity in cells, allowing cells to metabolize glucose more quickly rather than storing it as body fat. As a result, vegan diets have been shown to increase postprandial calorie burn by about 16%, up to 3 h after consuming a meal.^26^ A 2007 study showed that the weight loss caused by a short-term dietary intervention with a plant-based diet was partially maintained over 2 years of follow-up.^27^

The reductions in mean fat intakes, saturated fat and cholesterol intakes in the intervention group were similar to those seen with a plant-based diet in previous studies.^23, 28^ The reductions in circulating levels of total and LDL cholesterol observed with plant-based diets are caused by the absence of animal fat, as well as by the lipid-lowering effect of certain plant-based foods,^29^ and are typically greater than those seen with more moderate diets.^30^ Low-fat vegetarian diets showing decreases in HDL cholesterol are not associated with poor cardiovascular health in observational studies,^31, 32^ and have been shown to improve atherosclerotic lesions and cardiac events despite lowering of HDL cholesterol in randomized controlled trials.^33, 34^ Among individuals with type 2 diabetes, a plant-based diet improves glycemic control and reduces body weight and plasma lipid concentrations.^15^

Strengths of the study include a geographically diverse population, the use of a simple intervention that is highly reproducible in other corporate locations and sufficient statistical power to demonstrate significant changes. Limitations of the study were underrepresentation of males for reasons that are not clear and lack of the data on physical activity. The participants were asked not to alter the exercise patterns during the study. We are not sure whether they followed the recommendation. Participants were self-selected and represent a small fraction of the total workforce, in part because normal-weight, non-diabetic individuals were excluded. However, it is not necessary to engage an entire employee group in health promotion activities in order to achieve substantial economic benefits, because health risks and medical expenditures tend to be concentrated in a small population of employees.^35^ There was an attrition rate of 34% in the intervention group. However, an intention-to-treat analysis (in which dropouts were deemed to have had no change from baseline) and an analysis for study completers yielded similar results. Recruitment fell short of the sample size called for by the power analysis. Nonetheless, significant results were achieved for key variables, suggesting that the power analysis may have provided an overestimation of sample size. Although our study establishes the important finding that a large corporate setting can indeed implement a plant-based diet intervention in widely diverse geographical locations and can achieve clinically important results in a 4-month period, the long-term sustainability is a separate issue. However, a similar intervention was sustained over a 2-year period in a study by the current investigators.^26^

In summary, a simple nutrition intervention at the place of employment, tested in widely divergent regions of the United States, yielded significant improvements in indicators associated with medical risk. Further studies should explore means of implementing nutritional interventions widely, particularly for individuals at greatest risk. This nutrition program can easily be implemented in large corporate worksites to conduct further studies exploring the effects of diet on other sources of disability and revenue loss such as depression, anxiety and work productivity. These findings can be used to encourage worksites to offer low-fat plant-based options in their cafeterias as well as offering a weekly, ongoing nutrition education group, an online discussion board and other forms of support for staff and employees.

## Figures and Tables

**Table 1:** Baseline characteristics by group assignment

*Characteristics*	*Intervention (*n=*142)*	*Control (*n=*149)*	P*-value*^a^
Age, years (s.d.)	44.3 (15.3)	46.1 (13.6)	0.29

*Gender n* (%)			
Men	32 (23%)	18 (12%)	0.02
Women	110 (77%)	132 (88%)	

*Race n* (%)			
White	88 (62%)	98 (65%)	0.30
Black	34 (24%)	41 (27%)	
Asian	10 (7%)	5 (3%)	
Other	10 (7%)	6 (4%)	

*Ethnicity n* (%)			0.17
Hispanic	16 (11%)	10 (7%)	
Non-Hispanic	126 (89%)	140 (93%)	

*Occupation n* (%)			0.36
Sales/service	90 (63%)	106 (71%)	
Supporting staff	34 (24%)	25 (17%)	
Professional	7 (5%)	10 (7%)	
Other	11 (8%)	9 (6%)	

a *P*-values refers to *χ*^2^ square test for categorical variables and *t*-test for continuous variable for differences between groups.

**Table 2:** Macronutrient changes by group assignment

	*Intervention group (*n=*119)*			*Control group (*n=*135)*			*Between-group difference in change score, mean, unadjusted*^a^ *(95% CI)*	Between-group difference in change score, mean, adjusted^*^ *(95% CI)*	P-*value*^b^
	*Baseline mean*	*18-week mean*	*Within-group difference*	*Baseline mean*	*18-week mean*	*Within-group difference*			
Energy (kcal)	1887 (65)	1623 (59)	−264.6 (65.6)^**^	1727.5 (50.3)	1690.7 (58.4)	−36.8 (58)	−228(−400 to −56.1)^****^	−130 (−281 to 20.4)	0.09
Protein, % of total energy	15.7 (0.37)	14.6 (0.41)	−1.2 (0.4)^***^	16.5 (0.4)	17.0 (0.5)	0.5 (0.5)	−1.7 (−2.9 to −0.4)^****^	−12.9 (−19.6 to −6.4)	<0.001
Total Fat, % of total energy	35.1 (0.9)	31.0 (1.0)	−4.1 (0.9)^**^	37.2 (0.7)	36.8 (0.7)	−0.38 (0.86)	−3.7 (−6.2 to −1.2)^***^	−15.2 (−22.7 to −7.6)	<0.001
Saturated fat, % of total energy	11.3 (0.4)	8.5 (0.5)	−2.8 (0.4)^**^	12.0 (0.4)	11.6 (0.4)	−0.36 (0.45)	−2.4 (−3.7 to −1.2)^**^	−6.7 (−9.7 to −3.7)	<0.001
Carbohydrate, % of total energy	50.1 (1.1)	56.6 (1.3)	6.4 (1.2)^**^	47.6 (0.9)	47.5 (0.9)	−0.07 (0.9)	6.5 (3.5 to 9.5)^**^	16.9 (−6.9 to 40.9)	0.16
Cholesterol (mg)	244.0 (18.8)	136.3 (18.1)	−107.7 (21.9)^**^	240.5 (15.3)	226.6 (18.6)	−13.9 (19.6)	−93.8 (−151.6 to −36)^***^	−92 (−141.5 to −42.6)	<0.001
Fiber (g)	19.3 (1.2)	22.9 (1.3)	3.5 (1.3)^***^	16.0 (0.7)	16.8 (0.8)	0.74 (0.72)	2.8 (−0.1 to 5.7)	4.6 (1.9 to 7.2)	0.001

Abbreviations: CI, confidence interval.

All values mean ±s.e.m.

*P-value for analysis adjusted for gender, baseline value and medication change.

**P<0.001.

***P<0.01.

****P<0.05.

a Unadjusted (from *t*-test)

b Gender, baseline value and cluster adjusted (analysis of covariance).

**Table 3:** Changes in clinical variables by group assignment among all participants

*Clinical measure*	*Intervention (*n=*142)*			*Control (*n=*149)*			*Between-group difference in change score, mean, unadjusted*^a^ *(95% CI)*	*Between-group difference in change score, mean, adjusted*^b^*(95% CI)*	P-value^*^
	*Baseline mean*	*18-week mean*	*Within-group difference*	*Baseline mean*	*18-week mean*	*Within-group difference*			
Weight (kg)	96.5 (1.9)	93.6 (1.9)	−2.9 (0.34)^**^	96.4 (1.9)	96.4 (1.9)	−0.06 (0.33)	−2.9 (−2.0 to −3.9)^**^	−2.8 (−3.8 to −1.8)	<0.001
BMI (kg/m^2^)	34.7 (0.6)	33.7 (0.6)	−1.04 (0.1)^**^	35.3 (0.7)	35.3 (0.7)	−0.01 (0.1)	−1.1 (−1.4 to −0.7)^**^	−1.0 (−1.4 to −0.7)	<0.001
Total cholesterol (mg/dl)	186.3 (3.4)	178.3 (3.1)	−8.0 (2.1)^**^	188.9 (3.2)	188.9 (3.0)	−0.01 (1.5)	−8.0 (−13.1 to −2.9)^***^	−8.5 (−13.1 to −3.8)	<0.001
LDL cholesterol (mg/dl)	107.9 (2.8)	99.8 (2.6)	−8.1 (1.8)^**^	108.5 (2.8)	107.6 (2.6)	−0.9 (1.4)	−7.2 (−11.8 to −2.7)^***^	−7.2 (−11.4 to −3.0)	<0.001
HDL cholesterol (mg/dl)	53.7 (1.4)	52.0 (1.3)	−1.8 (0.6)^***^	56.7 (1.2)	57.7 (1.2)	0.9 (0.6)	−2.7 (−4.4 to −1.1)^***^	−3.1 (−4.6 to −1.6)	<0.001
Total:HDL cholesterol ratio	3.7 (0.1)	3.7 (0.1)	−0.05 (0.04)	3.5 (0.1)	3.4 (0.1)	−0.07 (0.03)	0.02 (−0.1 to 0.13)	0.02 (−0.1 to 0.12)	0.77
Triglycerides (mg/dl)	123.6 (5.5)	133.5 (5.6)	9.9 (3.8)^****^	119.4 (5.3)	118.0 (5.0)	−1.4 ((3.3)	11.4 (1.4 to 21.3)^****^	10.7 (1.5 to 20)	0.02
Systolic blood pressure (mm Hg)	126.5 (1.2)	124.8 (1.1)	−1.7 (0.7)^****^	127.2 (1.3)	124.5 (1.1)	−2.8 (0.9)^***^	1.2 (−1.1 to 3.4)	0.9 (−1.1 to 2.9)	0.38
Diastolic blood pressure (mm Hg)	82.3 (0.8)	80.0 (0.8)	−2.4 (0.6)^**^	81.6 (0.8)	79.4 (0.7)	−2.0 (0.5)^**^	−0.2 (−1.7 to 1.4)	0.4 (−0.9 to 1.8)	0.54
Hemoglobin A1c, % (*n*=21 diet group; control group *n*=22)	7.54 (0.42)	6.94 (0.40)	−0.60 (0.17)^****^	7.05 (0.32)	7.13 (0.34)	−0.08 (0.09)	−0.68 (−0.29 to −1.1)^****^	−0.7 (−1.2 to −0.2)	0.004

Abbreviations: BMI, body mass index; CI, confidence interval; HDL, high-density lipoprotein; LDL, low-density lipoprotein.

All values mean±s.e.m. unless otherwise specified.

^*^P-value for analysis adjusted for gender, cluster, medication change and baseline value.

^**^P<0.001.

^***^P<0.01.

^****^P<0.05.

a Unadjusted (from *t*-test)

b Gender, cluster, medication change and baseline value adjusted (analysis of covariance).

**Table 4:** Changes in clinical variables by group assignment among program completers

*Clinical measure*	*Intervention (*n=*96)*			*Control (*n=*119)*			*Between-group difference in change score, mean, unadjusted*^a^ *(95% CI)*	*Between-group difference in change score, mean, adjusted*^b^ *(95% CI)*	P-value^*^
	*Baseline mean*	*18-week mean*	*Within-group difference*	*Baseline mean*	*18-week mean*	*Within-group difference*			
Weight (kg)	93.3 (2.1)	89 (2.0)	−4.3 (0.4)^**^	93.5 (1.9)	93.4 (1.9)	−0.08 (0.4)	−4.3 (−3.2 to −5.5)^**^	−4.3 (−5.5 to −3.1)	<0.001
BMI (kg/m^2^)	33.5 (0.6)	32 (0.6)	−1.5 (0.2)^**^	34 (0.6)	34 (0.6)	−0.02 (0.1)	−1.6 (−1.1 to −1.9)^**^	−1.6 (−2.0 to −1.1)	<0.001
Total cholesterol (mg/dl)	189 (4.3)	176 (4.2)	−13.7 (3.5)^**^	191 (3.5)	189 (3.5)	−1.3 (2.3)	−12.3 (−4.3 to −20.3)^***^	−12.6 (−20.1 to −4.9)	0.001
LDL cholesterol (mg/dl)	109 (3.7)	96 (3.4)	−13.0 (2.8)^**^	110 (3.2)	108 (3.0)	−1.7 (1.9)	−11.3 (−17.8 to −4.8)^***^	−11.1 (−17.2 to −5.1)	<0.001
HDL cholesterol (mg/dl)	55 (1.8)	51 (1.7)	−3.3 (1.1)^***^	56 (1.4)	56.7 (1.3)	0.7 (0.9)	3.9 (−6.6 to −1.3)^***^	−4.1 (−6.6 to −1.6)	0.001
Total:HDL cholesterol ratio	3.7 (0.1)	3.7 (0.1)	−0.07 (0.06)	3.6 (0.9)	3.5 (0.8)	−0.08 (0.05)	0.01 (−0.1 to 0.1)	0.04 (−0.1 to 0.19)	0.63
Triglycerides (mg/dl)	129 (7.0)	143 (7.1)	13.9 (5.6)^****^	127 (6.28)	124 (5.9)	−2.9 (4.3)	16.8 (3.1 to 30.6)^****^	14.1 (1.3 to 27)	0.03
Systolic blood pressure (mm Hg)	127 (1.6)	124 (1.4)	−2.6 (1.0)^****^	127 (1.4)	123 (1.0)	−3.6 (1.1)^***^	0.9 (−2.1 to 4.1)	0. 8 (−1.9 to 3.1)	0.60
Diastolic blood pressure (mm Hg)	83 (1.1)	79 (1.0)	−3.5 (0.8)^**^	81 (0.9)	79 (0.8)	−2.6 (0.7)^**^	−0.8 (−2.9 to 1.1)	0.4 (−2.2 to 1.4)	0.64
Hemoglobin A1c, % (*n*=17 diet group; control group *n*=18)	7.52 (0.49)	6.78 (0.44)	−0.74 (0.19)^***^	7.03 (0.36)	7.13 (0.38)	−0.10 (0.12)	−0.84 (−0.37 to −1.1)^***^	−0.7 (−1.2 to −0.3)	0.003

Abbreviations: BMI, body mass index; CI, confidence interval; HDL, high-density lipoprotein; LDL, low-density lipoprotein.

All values mean±s.e.m. unless otherwise specified

^*^P-value for analysis adjusted for gender, cluster and baseline value.

^**^P<0.001.

^***^P<0.01.

^****^P<0.05.

a Unadjusted (from *t*-test).

b Gender, cluster and baseline value (analysis of covariance).

## Notes

Dr Neal Barnard gives lectures and writes books on the subject of plant-based diets and receives occasional honoraria and royalties therefrom. The remaining authors declare no conflict of interest.
